# The Over-expression of the Plastidial Transglutaminase from Maize in *Arabidopsis* Increases the Activation Threshold of Photoprotection

**DOI:** 10.3389/fpls.2016.00635

**Published:** 2016-05-10

**Authors:** Nikolaos E. Ioannidis, Dimitris Malliarakis, Josep M. Torné, Mireya Santos, Kiriakos Kotzabasis

**Affiliations:** ^1^Department of Biology, University of CreteHeraklion, Greece; ^2^Department of Molecular Genetics, Center for Research in Agricultural GenomicsBarcelona, Spain

**Keywords:** polyamines, tolerance, photosynthesis, plant fitness, antenna regulation

## Abstract

Plastidial transglutaminase is one of the most promising enzymes in chloroplast bioenergetics due to its link with polyamine pathways and the cross talk with signals such as Ca^2+^ and GTP. Here, we show the effect of the increase of transglutaminase activity in *Arabidopsis* by using genetic transformation techniques. These lines fulfill their biological cycle normally (normal growth in soil, production of viable seeds) and show a relatively mild increase in transglutaminase activity (127%). These overexpressors of transglutaminase (OE TGase) have an extended stroma thylakoid network (71% higher number of PSIIβ centers), similar chlorophyll content (-4%), higher linear electron flow (+13%), and higher threshold of photoprotection activation (∼100%). On the other hand OE TGase showed a reduced maximum photochemistry of PSII (-6.5%), a smaller antenna per photosystem II (-25%), a lower photoprotective “energization” quenching or qE (-77% at 490 μmol photons m^-2^ s^-1^) due to a higher threshold of qE activation and slightly lower light induced proton motive force (-17%). The role of the polyamines and of the transglutaminase in the regulation of chemiosmosis and photoprotection in chloroplasts is discussed.

## Introduction

Photosynthesis is one of the most important biochemical processes in the plant cell producing via light reactions energy (in terms of ATP), oxygen and reducing power (in terms of NADPH; Niyogi, 1999; Ioannidis et al., 2012b; Ioannidis and Kotzabasis, 2014). The processes of the thylakoid or “coupling” membrane need rapid auto-regulation (response within seconds). The danger for the coupling membrane is to lose functionality and structural integrity due to imbalances in regulation of redox and ionic processes. Hence, the photosynthetic apparatus has evolved self-activated and self-regulatory loops that assure efficiency both at low and high light as well as during rapid fluctuations of light intensity.

Some of the main regulatory loops that play key roles in plant bioenergetics are proton motive force (*pmf*)-dependent. The first regulatory loop that received considerable interest the last decade is “energization” quenching or energy-dependent exciton quenching (qE; Ruban et al., 2007). This loop is activated by the osmotic (i.e., ΔpH) component of *pmf* and confers protection to the plant by converting excess photonic energy into heat (Müller et al., 2001; Ioannidis and Kotzabasis, 2015). A second regulatory mechanism is the regulation of the amplitude of lumen electric field (Δψ). This electric field powers ATP synthesis, activates the protein channel that increase Cl^-^ in lumen compensating charge changes (Schoenknecht et al., 1988) and regulates the rate of linear electron flow (LEF) at the step of cytb_6_f (Hope, 2000). A third loop is the regulation of LHCII affinity either to PSII or to PSI via a redox activated kinase that phosphorylates the stroma exposed surface of LHCII known as state transitions (Haldrup et al., 2001).

Most of the aforementioned loops are satisfied by thylakoidal *pmf* and its partitioning in ΔpH and Δψ. In this context, recently discovered players (i.e., polyamines) are among the basic means of the plant cell to coordinate photosynthetic subcomplexes, and consequently define to a great extent the yield and protection limits of photosynthesis via *pmf* modulation. Therefore biosynthetic, catabolic and regulatory processes that produce, oxidize, or attach polyamines gain considerable merit in plant cell physiology.

Biochemistry of polyamines is quite simple, but the regulation of their titer is strikingly complex. More particularly the main polyamines putrescine (Put), spermidine (Spd), and spermine (Spm) derive in chloroplasts from arginine, have a relatively small molecular weight (88, 145, 202) and a maximum charge of +2, +3, and +4, respectively (Ioannidis et al., 2006; Del Duca et al., 2014). On the other hand arginine decarboxylase (ADC EC. 4.1.1.19) in plants is located in thylakoids of chloroplasts (Borrell et al., 1995; Bortolotti et al., 2004), is induced by stress like osmotic stress (Flores and Galston, 1982; Capell et al., 2004) or high salinity (Chattopadhyay et al., 1997) and has a K_m_ ranging from 0.03 mM in oat to 1.73 mM in Lathyrus (Galston and Sawhney, 1990). Spm can be converted back to Put via the action of polyamine oxidases (Moschou et al., 2008). In addition, free polyamines can be conjugated reversibly to phenolics and proteins (Serafini-Fracassini et al., 1988; Del Duca et al., 2014) via covalent bonds or bind firmly to negative surfaces (RNAs, proteins, lipids) via coulombic forces (Igarashi and Kashiwagi, 2010). Polyamine binding to plastidial proteins is the least understood process and the corresponding enzymes (plastidial transglutaminases) were relatively recently cloned (Villalobos et al., 2004). To the following we will briefly review the basic links between polyamines and photosynthesis as well as our current understanding of tranglutaminase role in chloroplasts.

Polyamines function both as organic cations and as permeant buffers. Their cationic effects increase stacking of thylakoids *in vitro* (Ioannidis and Kotzabasis, 2007), mimic the stimulatory effect of monovalent and divalent inorganic cations *in vitro* (Ioannidis et al., 2006) and alter the secondary structure of photosystems (Bograh et al., 1997; Beauchemin et al., 2007; Yaakoubi et al., 2014). For a recent review see (Hamdani et al., 2011). Positive charges of polyamines help them interact with chloroplastic DNA, RNA and ribosomes, and with negative charges of protein surfaces such as LHCII (Tsiavos et al., 2012). The buffering effect of polyamines is based on ion trapping. Free forms accumulate in lumen during proton release from photosynthesis and are trapped as they get protonated (Ioannidis et al., 2012a). Polyamine accumulation in the lumen promotes increases in ATP *in vitro* (Ioannidis et al., 2006) and electric field *in vivo and in vitro* (Ioannidis et al., 2012a).

Transglutaminases (TGases R-glutaminylpeptide-amine γ-glutamyltransferase; E.C. 2.3.2.13) are rather overlooked factors of the thylakoid system. These enzymes catalyze post-translational modification of proteins by establishing ε-(γ-glutamyl) links and covalent conjugation of polyamines (Lorand and Graham, 2003; Serafini-Fracassini and Del Duca, 2008). In plants, using *Helianthus tuberosus* isolated leaf chloroplasts, it was shown that some antenna proteins of the photosystems (LHCII, CP29, CP26, and CP24) were substrates of TGase (Serafini-Fracassini et al., 1988; Del Duca et al., 1994). TGase activity was shown to be light sensitive, affected by hormone deprivation and with a light/dark rhythm (Bernet et al., 1999). Immunogold localization of transglutaminase in different maize cell types using anti *Helianthus tuberosus* TGase antibody showed that the enzyme is specifically localized in the chloroplast grana-appressed thylakoids, close to LHCII, its abundance depending on the degree of grana development (Villalobos et al., 2001). An important step in elucidating the role of plastidial TGase was the isolation for the first time in plants of two related complementary maize cDNA clones, tgz15 and tgz21, encoding active maize TGase (TGZ; Villalobos et al., 2004). Their expression was dependent on length of light exposure, indicating a role in adaptation to different light environmental conditions, including natural habitats (Pintó-Marijuan et al., 2007). Taking into account all the described results, it has been hypothesized that TGases are implicated in the photosynthetic process (Villalobos et al., 2004; Pintó-Marijuan et al., 2007; Serafini-Fracassini and Del Duca, 2008). Proteomic studies indicate that maize chloroplastic TGase is a peripheral thylakoid protein forming part of a specific PSII protein complex which includes LHCII, ATPase and PsbS proteins (Campos et al., 2010). First evidence for a role of plastidial TGase in the thylakoids 3D architecture comes from tobacco over expressing maize TGase (Ioannidis et al., 2009). Transformed tobacco chloroplasts, with a fourfold increase of TGase activity, showed stroma thylakoid depletion and granum size increase. At the same time, as a consequence of increased TGase activity, antenna-associated polyamine content also increased and PSII with large absorption cross section accumulated in these plants (Ioannidis et al., 2012b). Two disadvantages of these model plants were the appearance of oxidative stress and amyloid-like protein inclusions in chloroplasts (Villar-Piqué et al., 2010) as well as the need to cultivate them on MS medium and sucrose. This fact made clear the need for new plant lines that will grow autotrophically and have a significant but not enormous high TGase activity. More recently, a rice transglutaminase gene *tgo* and its recombinant protein TGO has been identified and characterized. This rice TGase was immunolocalized in the chloroplast, although not exclusively (Campos et al., 2013). Moreover, some LHCII antenna proteins as well as ATPase and some PSII core proteins were identified as TGO associated proteins. Plastidial TGase in rice is light dependent, regulated by the illumination period, and related to plastidial proteins implicated in photoprotection and in thylakoid electrochemical gradient (Campos et al., 2014). In this contribution, we present the first lines of *Arabidopsis* plants that overexpress maize TGase, named Col-0 tgz15 or OE TGase.

## Materials and Methods

### Plant Material

A protocol of requirements adapted to *Arabidopsis* plants development and controlled by CRAG Greenhouses Services were established to plants support. The wild type seeds of *Arabidopsis thaliana*, Columbia and *Arabidopsis* transformed plants were multiplied in the same environmental conditions. All plants have been cultivated in a CRAG confined chamber services [facilities for genetically modified organisms (GMOs)] with the adequate safety to prevent material release from containment within the greenhouses or growth chambers.

### Plant Transformation

*Arabidopsis thaliana* Columbia plants were transformed with the *Agrobacterium tumefaciens* strain C58C1 that carries the Ti disarmed vector, using the floral dip infiltration method. For the phenotype of transformed plants please see **supplementary Figure 1**. A modified binary vector was used for transformation. The pGV2260 vector includes a fragment of the pBR322 plasmid, substituting the T-DNA. For the selection of transformed lines carbeniciline was used. The plasmid-TL region is substituted by a carbeniciline resistance gene. The maize transglutaminase *tgz15* cDNA [EMBL Data Library accession numbers: AJ421525 (TGZ15)] was cloned into the plasmid pGreen 0029. The *tgz15* gene is preceded by two 35S constitutive promoters and followed by a CaMV terminator, all obtained from pJIT60. Both 35S promoters were excised from pJIT60 with SacI and HindIII and CaMV terminator was excised with EcoRI and EcoRV. The promoter addition to the plasmid pGreen0029 was realized between SacI and BamHI; the *tgz15* insert was added between BamHI and EcoRI and the terminator CaMV between EcoRI and Eco RV.

**FIGURE 1:**
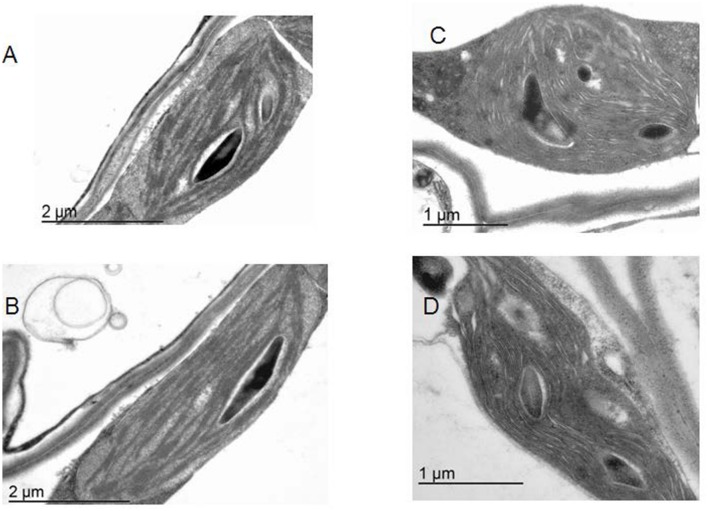
**The architecture of plastids.** Electron microscopy for WT *Arabidopsis* line **(A,B)** and the lines overexpressing TGase **(C,D)**.

### Selection of Transformed *Arabidopsis*

The pGreen 0029 vector used in tgz15 cloning confers kanamycin resistance. In order to select tgz15 transformed seedlings, sterilized seeds were cultured in MS medium supplemented with 25 μg/mL kanamycin and 100 μg/mL cefotaxime using Petri dishes. Kanamycin is the selective agent from transgenic plants and the cefotaxime allows elimination of residual *Agrobacterium*. Petri dishes with seeds are stratified a minimum of 2 days at 4°C in dark and during 7 days cultured at 22°C under long day photoperiod (16 h illumination). Surviving seedlings are placed in humid substrate pots, at 22°C and short day photoperiod (8 h illumination). When the leaflets of plants have 2 cm diameter, pots can be maintained in greenhouse until the siliques formation and seed stocking. Previous to the genetic analyses, to confirm the gene insertion, plant selection in kanamycin medium is repeated for some plant/clones, to assure their homogeneity.

### DNA Extraction, PCR Reactions, and *tgz* Gene Incorporation

For leaf DNA extraction, the Quiagen cDNA Easy plant mini kit was used following the manufacturer’s instructions. The PCR conditions were standardized as follows: 2 min at 95°C for initial denaturation: 40 cycles of 30 s at 95°C, 45 s at 55°C, 150 s at 75°C and 7 min at 72°C for final extension. The PCR TaKaRa Ex Taq ^TM^ kit was used (dNTPs 2.5 mM, Taq polymerase 0.5 μl, and 5x buffer). The oligonucleotides used as primers to detect the presence of the insert were as follows:

Forward primer: 5′ ATACAACTATGCTTATGATGCTGG CACG 3′
Reverse primer: 5′ TATTTGTCTGCTCAACAAATGTGC ATG 3′.

These primers were designed in a DNA region of the maize *tgz* sequence that did not have any significant homology with the database DNA *Arabidopsis* sequences. The selected DNA region, of 371 bp, was near the *tgz* 3′ end.

To confirm the presence of the expected amplified DNA in the transformed plants, the PCR products were run on a 1.2% agarose gel and visualized. Results were compared to *tgz-*tobacco DNA overexpressor, used as the positive control in the same PCR experiment, to confirm the same *tgz* band. The selected PCR amplified *tgz*-DNA bands were extracted and sequenced to confirm the *tgz* gene incorporation (**Supplementary Figure S1**).

Seeds of transformed plants are available in NASC (Name: Col-0 tgz15; NASC ID: N799986).

### Electron Microscopy

The methodology used to observe the ultrastructure of *Arabidopsis* plants (wild type and transformed plants) is similar to procedures used in tobacco *tgz* transformed plants (Ioannidis et al., 2009).

### Spectroscopic Assays

The methods for measuring extents of energization qE, rates of LEF, and the relative extents of *pmf* components were as described in Kanazawa and Kramer (2002) except that a newly developed instrument was used. This instrument was based on the non-focusing optics spectrophotometer (NoFOSpec; Kramer and Sacksteder, 1998) but has been modified to allow near-simultaneous measurements of absorbance changes at four different wavelengths, by aiming four separate banks of light-emitting diodes (HLMP-CM15, Agilent Technologies, Santa Clara, CA, USA), each filtered through a separate 5 nm bandpass interference filter (Omega Optical, Brattleboro, VT, USA), into the entrance of a compound parabolic concentrator (Hall et al., 2013). Each complete set of three pulses was deconvoluted by using the procedure described previously (Avenson et al., 2004; Ioannidis et al., 2012a) to obtain estimates of electrochromic shift (ECS). The instrument was also used to measure changes in chlorophyll *a* fluorescence yield by using the 520 nm light-emitting diode bank as a probe beam, as described previously. Saturation pulses (>7,000 μmol photons m^-2^ s^-1^ photosynthetically active radiation) were imposed by using light from the two red actinic LEDs, filtered through heat-absorbing glass. Actinic light was filtered out by using an RG-695 Schott glass filter (Ioannidis et al., 2014). Saturation pulse-induced fluorescence yield changes were interpreted as described in Genty et al. (1989). Non-photochemical quenching of Chl fluorescence (NPQ) was determined in samples exposed to actinic light of selected light intensities ranging from 64 to 750 μmol photons m^-2^ s^-1^. Samples were adapted in the dark for at least 10 min prior to measurement. The qE component of NPQ was calculated from the saturation pulse-induced maximum fluorescence yields during steady-state illumination (Fm′) and 10 min (Fm″) after switching off the actinic light (Navakoudis et al., 2007). The NPQ-parameter was calculated according to the equation: NPQ = (Fm–Fm′)/Fm′and qE according to the equation qE = (Fm″–Fm′)/F′ (Bilger and Björkman, 1990).

### *In Vivo* Measurements of Proton Flux and *pmf* Characteristics

This work and analysis are made possible by newly introduced techniques that allow us to non-invasively probe the “proton circuit” of photosynthesis. For the theoretical framework for these methods see Avenson et al. (2004) and Ioannidis et al. (2012a) and refs therein. These techniques take advantage of the ECS (sometimes called ΔA520 or ΔA518) of certain carotenoid species that naturally occur in the thylakoid membranes. The ECS is a linear indicator of changes in transthylakoid Δψ and is particularly useful for our studies because it responds to the transthylakoid movement of protons, as well as other charged species. We probed the ECS by using a previously described technique called dark-interval relaxation kinetic analysis (Sacksteder et al., 2000), in which steady-state photosynthesis is perturbed by short (up to 0.5 s), dark intervals, allowing the photosynthetic apparatus to relax in ways that reveal information about the system in the steady state. The amplitude of the light–dark ECS signal (ECSt) parameter was obtained by taking the total amplitude of the rapid phase of ECS decay from steady state to its quasistable level after ∼300 ms of darkness (Kanazawa and Kramer, 2002). For the deconvolution all traces were normalized to the initial dark value (i.e., before actinic) and then the following equation was used: ECS520 = A520 – 0.5 × A535 – 0.5 × A505 (Ioannidis et al., 2012a).

### Fluorescence Measurements *In Vivo*

For the fluorescence induction measurements, the portable Plant Efficiency Analyzer, PEA (Hansatech Instruments) was used as previously described (Kotakis et al., 2014). The method is based on the measurement of a fast fluorescent transient with a 10 μs resolution in a time span of 40 μs to 1 s. Fluorescence was measured at a 12 bit resolution and excited by three light-emitting diodes providing an intensity of 3000 μmol photons m^-2^ s^-1^ of red light (650 nm). For the estimation of the maximum quantum yield of PSII photochemistry the following equation was used Fv/Fm = (Fm–Fo)/Fm (Fm the maximal fluorescence of a dark adapted leaf and Fo the initial fluorescence). For the maximum capacity of quenching due to quinone pool (qPQ) the following equation was used qPQ = (Fm–F_30ms_)/(Fm–Fo) (Strasser et al., 2004). For the estimation of PSIIα and PSIIβ, DCMU inhibited electron transport after Q_A_ and analysis of the area closure was performed according to the method of Melis and Homann (1978) as modified in Ioannidis et al. (2009). More particularly, to estimate antenna heterogeneity, leaves were immersed in DCMU (50 μM) for 30 min in the dark. The half rise time of the *F*_V_ in the presence of DCMU was used as an estimate of effective antenna size of PSII (Cadoret et al., 2004; Ioannidis et al., 2009). The fraction of PSIIβ centers were calculated from the slope of ln[(Amx – At)/Amx] according to the method of (Anderson and Melis, 1983) with minor modifications (Ioannidis et al., 2009). Amx is the complementary area in the DCMU curves and At the area at time *t*.

### Fluorescence Measurements at 77 K

Low temperature (77 K) fluorescence spectra were recorded using a LS-50B spectrophotometer luminometer (Perkin Elmer) according to the manufacturer’s instructions. Samples were mounted in a special base emerged in liquid nitrogen and a stream of nitrogen gas excluded ice formation in the glass cuvette. For the blue shift (B shift) determination in **Table 1** the value of the emission peak was subtracted from 685 nm (standard emission peak of photosystem II).

**Table 1:** Comparison of structural and functional aspects of WT and OE TGase (*n* ≥ 3).

	WT	OE TGase
TGase activity (μmol mgprot^-1^h^-1^)	41155.08 ± 184.75	93741.68 ± 369.09ˆ*
Fo	496 ± 12.6	607 ± 35*
Fm	3051 ± 167	2734 ± 198*
Fv/Fm	0.83 ± 0.006	0.78 ± 0.003*
qPQ	0.16 ± 0.02	0.2 ± 0.013
F*_PSII_*/F*_PSI_*	2.48 ± 0.15	2.27 ± 0.036
B shift (from 685 nm)	0.675 ± 0.27	0.39 ± 0.08
Chls (mg cm^-2^)	12.54 ± 0.2	12.02 ± 0.3
Chl a/b	2.857 ± 0.09	2.842 ± 0.11
*PSIIa*	0.71 ± 0.02	0.50 ± 0.03*
*PSII*β	0.29 ± 0.02	0.50 ± 0.03*
Absorption cross section per PSII	5.08 ± 0.32	3.81 ± 0.03*
Δψ/*pmf* (at 366 μmol photons m^-2^ s^-1^)	0.704 ± 0.054	0.862 ± 0.029*
Polyamines in leaves (nmol g FW^-1^)	1115.2 ± 89	970.8 ± 73
qE sensitivity to *pmf* (slope **Figure 5A**)	0.588 ± 0.053	0.124 ± 0.011*


*^∗^Asterisks indicate significant differences at *p* ≤ 0.05.*

### Treatments of OE TGase Plants

For testing whether it is feasible to increase the relatively low qE and 535 nm scattering in OE TGase we performed three different experiments. We used high light intensity, supply of putrescine and low temperature. For the high light experiment, WT and OE TGase grown at 100 μmol photons m^-2^ s^-1^ were exposed for 15 min to 750 μmol photons m^-2^ s^-1^. For the low temperature experiments WT and OE TGase plants were transferred at 5°C overnight in the dark, then exposed to 500 μmol photons m^-2^ s^-1^ (at 5°C) and qE was estimated after 2 and 5 h of illumination (at 5°C). For the application of putrescine the roots of healthy WT and OE TGase plants were gently cleaned from soil and whole plants were mounted in small erlenmeyer flasks with cotton and parafilm sealing the flask. The solution (total volume 25 mL) of putrescine (free base) had a concentration of 3 mM and qE in intact leaves was estimated 4 h after application. The plants were illuminated during this 4 h period with 100 μmol photons m^-2^ s^-1^.

### Transglutaminase Activity

Transglutaminase activity was calculated by the hydroxamate method (Grossowicz et al., 1950).

### Polyamine Analysis by High Performance Liquid Chromatography (HPLC)

Polyamines were extracted as previously described and analyzed following the method of Kotzabasis et al. (1993). Briefly, for polyamine analysis leaf powder after liquid nitrogen was suspended in 1 N NaOH. A volume of 0.2 ml from the hydrolysate was mixed with 36% HCl in a ratio of 1:1 (v/v) and incubated at 110°C for 18 h. The hydrolysate was evaporated at 70–80°C. The dried products were re-dissolved in 0.2 ml of 5% (v/v) perchloric acid. To identify and estimate the polyamines, the samples were derivatized by benzoylation, as previously described (Sfichi-Duke et al., 2008). For this purpose, 1 ml of 2 N NaOH and 10 μl benzoylchloride were added to 0.2 ml of the hydrolysate and the mixture vortexed for 30 s. After 20 min incubation at room temperature, 2 ml of saturated NaCl solution were added to stop the reaction. The benzoylpolyamines were extracted three times into 2–3 ml diethylether; all ether phases collected and evaporated to dryness. The remaining benzoylpolyamines were redissolved in 0.2 ml of 63% (v/v) methanol and 20 μl aliquots of this solution were injected into the high performance liquid chromatography (HPLC) system for the polyamine analysis, as described previously (Sfichi-Duke et al., 2008). The analyses were performed with a Shimadzu Liquid Chromatography apparatus (LC-10AD) equipped with a SPD-M10A diode array detector (Shimadzu SPD-M10A) and a narrow-bore column (C18, 2.1 mm × 200 mm, 5 μm particle size Hypersyl, Hewlett-Packard, USA).

### Statistics

All experiments were performed three times. Statistical significance was checked with *t*-test (*p* < 0.05). Treatment of OE TGase (cold exposure, high light exposure and exogenous supply of putrescine) were performed a single time just to demonstrate that high levels of qE and light dependent absorbance changes at 535 nm occur in OE TGase.

## Results

### Structural and Architectural Differences

Overexpression of the plastidial transglutaminase of maize (TGZ) in *Arabidopsis* increased TGase activity almost 127% (**Table 1**). The genetically modified plants grow normally in soil and produce viable seeds. Their thylakoid network looks normal and their organelles seem swollen (**Figure 1**). The phenotype of OE TGase and WT mature plants is similar (**Supplementary Figure S2**).

**FIGURE 2:**
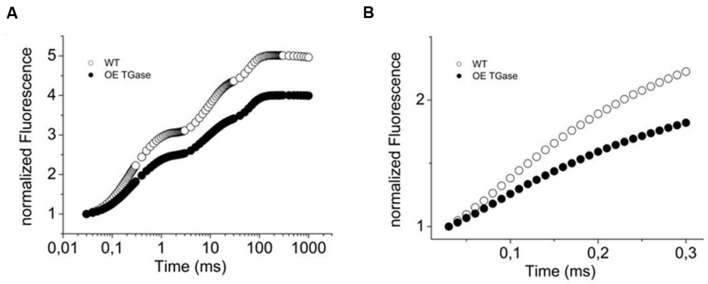
**Variable fluorescence (OJIP curve) of WT *Arabidopsis* (col) and over expressors of plastidial transglutaminase (OE TGase).** **(A)** Fluorescence induction curves normalized to Fo values for WT and OE TGase. **(B)** Variable fluorescence curves in DCMU inhibited leaves. Reaction centers close at a slower rate in OE TGase (i.e., lower values of fluorescence) indicating that the absorption cross section of PSII is smaller. For a quantitative estimation of antenna size see also **Table 1**.

**FIGURE 3:**
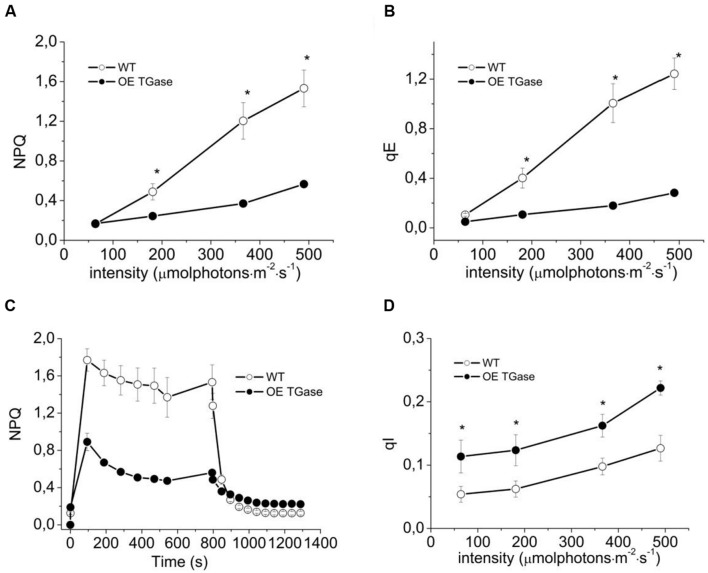
**Short term photoprotection mechanisms for intact WT (open symbols) and OE TGase (closed symbols).** **(A)** Non-photochemical quenching in intact plants exposed to four different light intensities. There is lower NPQ for OE TGase, which is more evident at the higher intensity. **(B)** Energization quenching (qE) in intact plants exposed to four different light intensities. **(C)** Typical traces of NPQ induction and relaxation kinetics at 490 μmol photons m^-2^ s^-1^. **(D)** Photoinhibition quenching qI under the conditions of **A,B**. Error bars denote standard error (*n* = 6) and asterisks statistically significant differences (*p* < 0.05).

Here, we present a functional and biochemical characterization of the OE line. Photosystem II functional organization was estimated by the fast (<0.5 s) signal of variable fluorescence and typical curves normalized to Fo values show that Fm is lowered in over expressors (**Figure 2A**). In experiments with inhibited PSII centers by DCMU, which in turn perform a single turnover cycle we estimated the absorption cross section of PSII (**Figure 2B** and **Table 1**). OE plants have 25% smaller antenna size in their PSII and a 20% higher capacity for quenching due to the pool of plastoquinone (**Table 1** index qPQ). Protective dissipation of the photonic energy was studied by standard spectroscopic tools (**Figure 3**). NPQ was significantly lower in OE (-73%) at high light (490 μmol photons m^-2^ s^-1^), but was similar (+2%) at low light intensities (64 μmol photons m^-2^ s^-1^) which mimicked growth intensity (i.e., 80–100 μmol photons m^2^ s^-1^; **Figures 3A,C**). The fast reversible (qE) and irreversible (qI) components of NPQ were estimated (**Figures 3B,D**). OE show 88% less qE than WT, while their qI remain at low values (∼0.2). There were only slight differences in the stoichiometries of PSII versus PSI as judged by the 8.5% decrease in the F_PSII_/F_PSI_ ratio, but a great increase (72%) of PSIIβ centers (**Table 1**). Total polyamines in leaves and Fv/Fm showed a mild but statistically significant decrease of 13 and 6%, respectively (**Table 1**). The Chl content and Chl *a*/*b* ratio was close to that of WT (**Table 1**). There is a smaller antenna per PSII (about 25%) in OE plants with similar fluorescence emission peak (B shift index). There is also a lower sensitivity of antenna to *pmf* in OE plants, that will be discussed in detail.

### Proton and Electron Circuit

**FIGURE 4:**
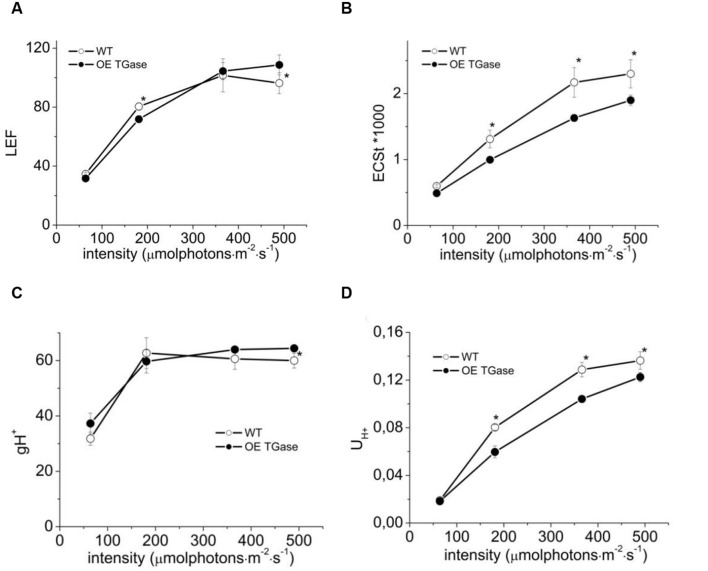
**Steady state photosynthesis in WT and OE TGase estimated in intact plants.** **(A)** Linear electron flow (LEF) at four different light intensities. **(B)** Light-induced proton motive force [estimated as electrochromic shift (ECS)] in thylakoids. **(C)** Apparent conductivity of the thylakoidal ATPase to protons (gH^+^). **(D)** Total proton flux from lumen to stroma (U_H+_). OE TGase show relatively small differences in the proton and electron circuit of photosynthesis. Error bars denote standard error (*n* = 6) and asterisks statistically significant differences (*p* < 0.05).

The proton and electron circuits of OE were studied by non-destructive means in intact plants (**Figure 4**). LEF was normally engaged for the range of intensities tested and OE showed a mild but statistically significant (13%) increase at high intensities (**Figure 4A**). A slight decrease in light-induced *pmf* (estimated as ECSt, **Figure 4B**) of OE was evident and ranged between 17 and 19%. On the contrary, apparent conductivity of thylakoidal ATPase to protons was 7–17% higher in OE for the range of light intensities checked (**Figure 4C**). The total proton efflux (U_H_^+^) from lumen to stroma was 4–10% lower (**Figure 4D**). The partitioning of *pmf* favored electric field (about 17% higher Δψ/*pmf* at 366 μmol photons m^-2^ s^-1^; **Table 1**).

### Antenna Down Regulation and Light Scattering

**FIGURE 5:**
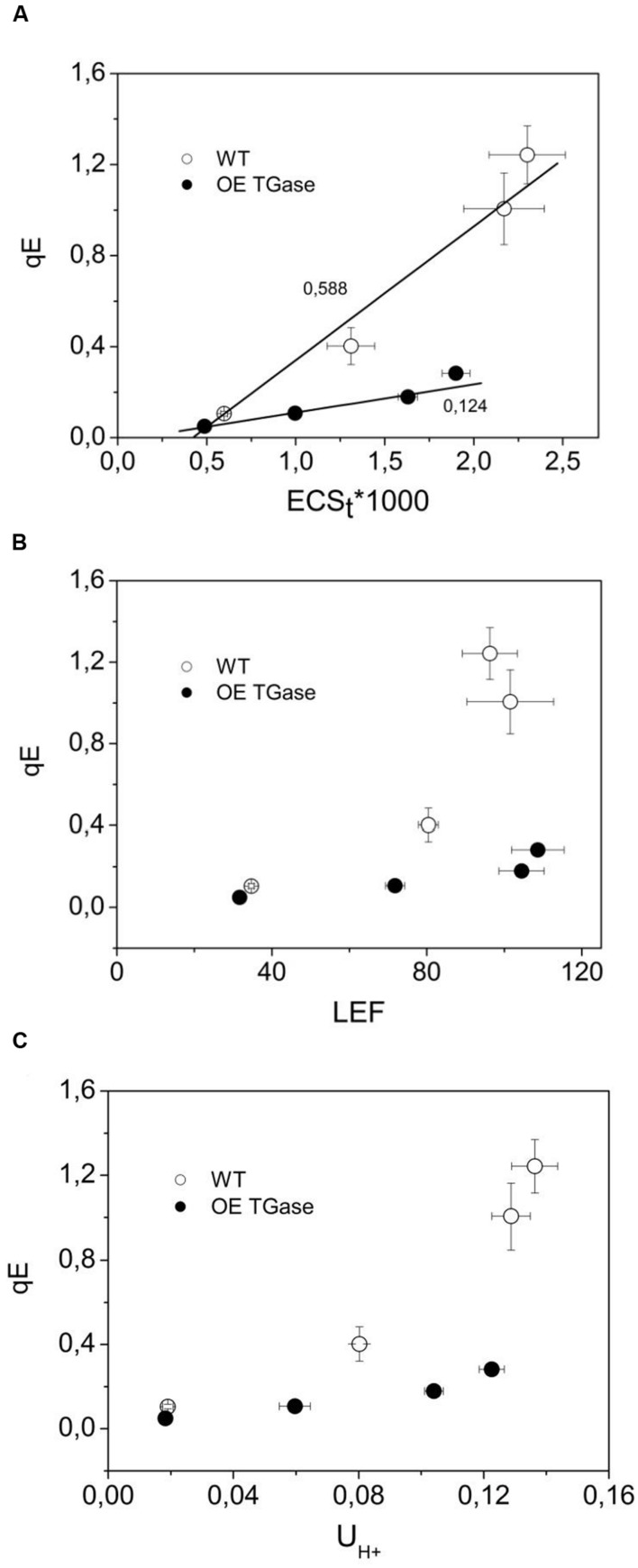
**The regulation of PSII antenna.** **(A)** The downregulation of the antenna in relation to the amplitude of *pmf*. **(B)** The downregulation of the antenna in relation to the amplitude of LEF. **(C)** The downregulation of the antenna in relation to proton efflux from lumen to stroma. The lower sensitivity of antenna downregulation (qE) to light induced proton motive force (ECSt) indicates that there is probably a higher buffering capacity and a higher Δψ/pmf in OE TGase thylakoids of intact leaves. By recording *in vivo* the decay of the ECS at three wavelengths and deconcoluting other processes we provide estimates of the higher Δψ/pmf in OE TGase (please see **Table 1**). Error bars denote standard error (*n* = 6).

**FIGURE 6:**
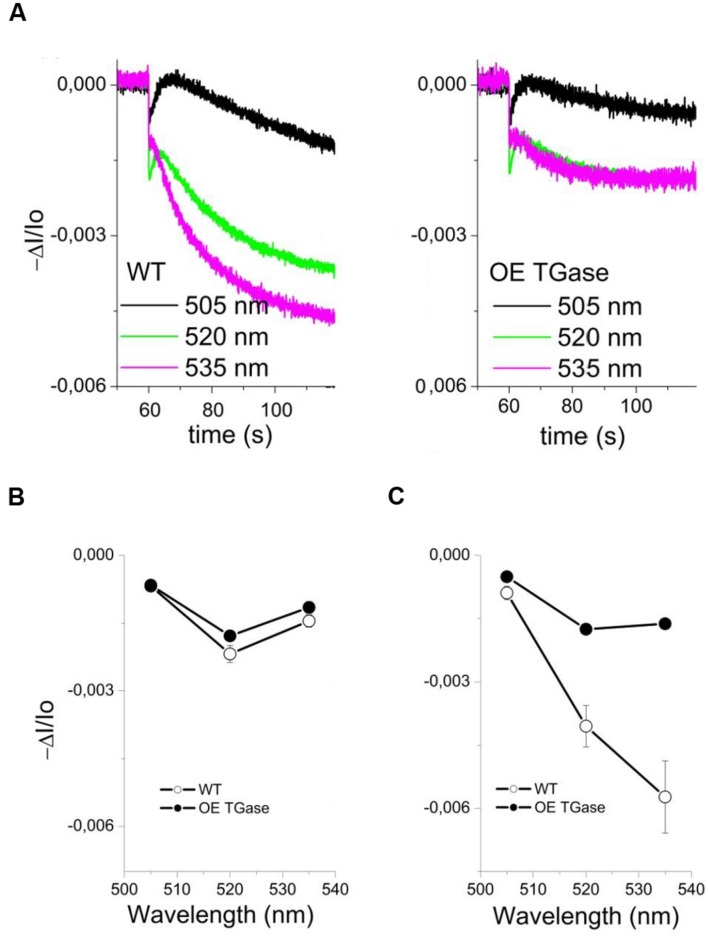
**Study of light scattering in intact WT and OE TGase leaves (kinetics, spectrum).** **(A)** Typical absorbance changes after cessation of actinic light (490 μmol photons m^-2^ s^-1^) and decay of the steady state signal in WT and OE TGase. The signal at 535 nm shows that OE TGase plants have marginal activation of its photoprotective mechanism. Absorbance changes at 535 nm are correlated with the qE response (see also low values of qE in **Figure 3B**). **(B)** The spectrum of *pmf* showing a clear peak at 520 nm both for WT and OE TGase. **(C)** The spectrum of light scattering showing a normal peak near 535 nm for WT, but a much smaller and more broad peak for OE TGase.

The sensitivity of antenna down regulation to light-induced *pmf* (**Figure 5A**) and to LEF (**Figure 5B**) decreases in OE. Similarly, the sensitivity of antenna down regulation to total proton efflux from lumen to stroma decreases in OE (**Figure 5C**). Photoprotective qE when activated is producing an increase of the 535 nm light scattering. In OE this signal is much lower than in WT (**Figure 6A**). OE TGase establish a light induced *pmf* with a normal spectrum peaking at around 520 nm (**Figure 6B**), but show a marginal light scattering component (**Figure 6C**).

### Threshold of qE Activation

**FIGURE 7:**
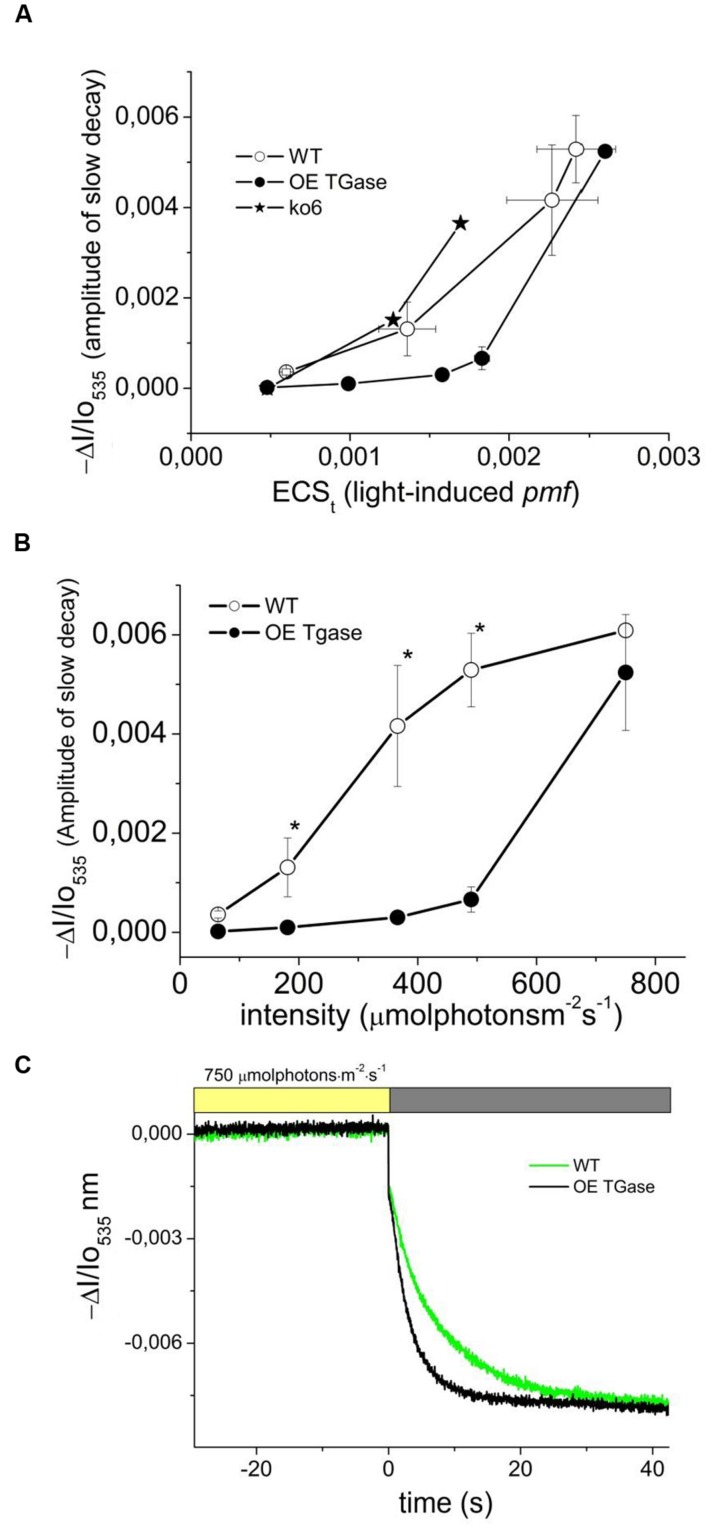
**Threshold of activation for the light scattering which is an index of photoprotection activation.** **(A)** Proton motive force probed by the rapid absorbance changes at 535 nm and energization of thylakoid membranes for intact WT and OE TGase plants probed by the amplitude of the slow absorbance changes at 535 nm. For comparison we plot data from experiments with antenna mutants under similar conditions (trace ko6). ko6 is a deletion mutant of CP24. **(B)** Light scattering as a function of light intensity. The results from absorbance spectroscopy are in line with the results from fluorescence spectroscopy (**Figure 3B**). Membrane energization (in terms of 535 nm amplitude) has a higher threshold of activation in OE TGase. **(C)** Typical absorbance changes after cessation of actinic light (750 μmol photons m^-2^ s^-1^) and decay of the steady state signal in WT and OE TGase. Thus at high light intensities we have similar amplitude of scattering changes, but OE TGase show much more rapid relaxation.

**FIGURE 8:**
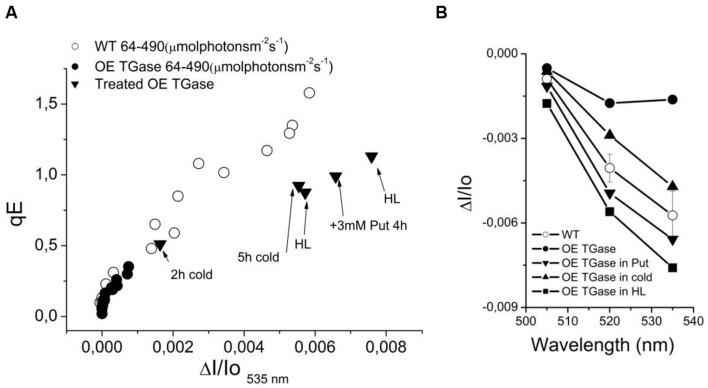
**The phenomenology of antenna down regulation.** **(A)** qE is linearly dependent on 535 nm signal. In OE TGase the signal at 535 nm is of very low amplitude (for the standard experimental conditions that used intensities from 64 up to 490 μmol photons m^-2^ s^-1^). The signal gets higher upon treatment with cold (see values for 2 and 5 h cold), with putrescine free base (see value for 3 mM Put) or exposure to 10-fold higher light than growth intensity (see values for HL). **(B)** The spectra of selected treatments (from 505 to 535 nm). The purpose of the treatments in OE TGase is to provide additional data pairs (for example in panel A qE and ΔI/Io_535_ nm values) for a broader range than the standard experiment. For example the standard experiment (64–490 μmol photons m^-2^ s^-1^) for OE TGase signal at 535 nm covers a range from 0 to 1 mAU unit, whereas by adding data from the different treatments one expands the range up to 8 mAU. For the WT the range is large enough and there is no need to introduce more data pairs.

In order to explore whether absorption change at 535 nm is not induced at 490 μmol photons m^-2^ s^-1^ due to decreased sensitivity or due to lack of functionality, we tried to increase it by several treatments (i.e., exposure to high light, cold, and putrescine). We estimated the precise threshold of activation in terms of *pmf* (**Figure 7A**) and light intensity (**Figure 7B**). This clearly shows that normal levels of activation are possible. A major difference is the light intensity that activates qE. Furthermore, upon activation light scattering decays faster in OE than in WT (**Figure 7C**). The relationship of qE versus the 535 nm signal shows similar activation curve with a slight insensitivity in the case of OE (**Figure 8A**). The exact slope was easily estimated for WT (for the conditions 64–490 μmol photons m^-2^ s^-1^), but this was not possible for OE due to its low signals. The slope for OE was based in pair of values (qE and A535) from additional experiments with stressors that could increase qE (**Figure 8A**). Furthermore, in order to test whether higher values of qE show normal scattering spectrum we checked the spectrum between 505 and 535 nm (**Figure 8B**). It is demonstrated that the spectrum of OE gets similar to WT after treatment with stressors. On the other hand the activation of the photoprotective qE is less sensitive to light induced proton motive force as judged by the amplitude of the slow decay at different levels of ECSt (**Figure 7A**).

## Discussion

A powerful tool to investigate the physiological role of an enzyme is the silencing of the coding gene. In the case of plastidial transglutaminase though, there are no available transformed plants with reduced levels of TGase activity. The best experimental line available so far was a transplastomic tobacco line which was overexpressing the plastidial transglutaminase from maize (TGZ; Ioannidis et al., 2009). This line was not autotrophic and its plastids accumulated large inclusion bodies. The progressive depletion of its Chl and stroma thylakoids was causing a quickly changing phenotype that hindered experimentation with these plants. Thus the need to cross check in a different model plant the role of plastidial TGase urged us to undertake the genetic modification of *Arabidopsis* with the maize TGase gene. The *Arabidopsis* TGase transformed lines grow normally and have an expanded thylakoid network without inclusion bodies (**Figure 1**). They also have a lower ratio of PSIIα/PSIIβ centers (**Table 1**) than WT. PSIIα centers have large antenna size and are reported to occur in grana regions (Melis and Homann, 1976; Melis, 1989; Kaftan et al., 1999; Kirchhoff et al., 2007; Ioannidis et al., 2009). Antenna size in PSIIβ is smaller and they are reported to occur in stroma lamellae (Melis, 1989; Kaftan et al., 1999; Kirchhoff et al., 2007; Ioannidis et al., 2009). The stable phenotype of the OE TGase plants allowed their functional characterization by biochemical and spectroscopic tools.

The higher activity of plastidial transglutaminase (127%) affected only marginally the maximal quantum yield of PSII (Fv/Fm ratio, **Figure 2A** and **Table 1**). The absorption cross section of PSII was 25% lower and the connectivity of PSII centers as judged by the amplitude of L band [index of energetic connectivity of reaction centers according to Oukarroum et al. (2007)] was similar (data not shown). Former studies suggested that the proteins of the antenna of PSII will hinder the pool of plastoquinone to access electrons produced by PSII (Tremmel et al., 2003). For this reason we checked whether OE plants show higher capacity of quenching due to the better access of the PQ pool. By estimating the parameter qPQ (Strasser et al., 1999), we show that OE has higher capacity for quenching via PQ pool (**Figure 2A** and **Table 1**). Hence, both parameters (absorption cross section of PSII and qPQ) agree that in OE photosystems II are organized and function differently than WT providing to PQ better access to PSII electrons. The decrease of the average absorption cross section of PSII antenna is consistent with the smaller ratio of PSIIα/PSIIβ centers.

In addition, smaller antenna results in less harvested photons when light flux is not saturating. This is the reason of lower LEF at low light (i.e., close to the growth intensity) which is about 10% lower in OE (**Figure 4A** values at 64 μmol photons m^-2^ s^-1^). Smaller antenna usually is beneficial for high light intensities (Polle et al., 2002). By exposing the lines to 5–10 times higher light intensity than the intensity experienced during growth we see a 13–15% higher LEF (**Figure 4A** and **Table 1**). On the other hand total proton flux from lumen to stroma (U_H+_) is estimated about 10% lower in OE, but if one takes into account that OE has 5% lower chlorophyll and the fact that U_H+_ takes information from ECSt, which is a Chl sensitive index, the actual difference in proton flux at 490 μmol photons m^-2^ s^-1^ should be less than 5%.

Light curves of NPQ show clearly that OE activates only a small fraction of maximal dissipation potential of the photosynthetic apparatus in *Arabidopsis* (**Figures 3A,C**). Low values of NPQ are established after 10 min of illumination and were set after higher values of NPQ evident during pre-steady state were de-activated by the plant (**Figure 3C**). This situation is not due to a defective photoprotective mechanism, but due to different sensitivity of qE to down regulation. In other words, both circuits of electrons and protons function normally (i.e., LEF and *pmf*), but qE is activated to a low level (**Figure 5**). Indication for a different threshold of qE activation is illustrated clearly by plotting qE as a function of *pmf* (**Figure 7A** and **Table 1** see qE sensitivity). Hence, OE shows 77% less qE because it has a 79% decrease of sensitivity to light induced *pmf*. The OE plants grow in low light (about 100 μmol photons m^-2^ s^-1^), but seem high light ready (i.e., perform better than WT upon exposure to high light intensities (500–750 μmol photons m^-2^ s^-1^). This adaptation to high light is in line with recent results showing that rice transglutaminase (TGO) is light dependent, regulated by the illumination period, and related to plastidial proteins specially those that are implicated in photoprotection and in thylakoid electrochemical gradient (Campos et al., 2014), as well as with former seminal works showing TGase catalyzing the modification of LHCII by polyamines in a light-dependent way (Della Mea et al., 2004). The role of polyamines and TGases in the acclimation/adaptation of the photosynthetic apparatus is also supported by data with the polyamine-deficient variant of *Dunaliela salina* (Dondini et al., 2001).

In order to delve into the role of plastidial TGase, we analyze the photoprotective mechanisms and their regulation in intact plants. A nice way to regulate the qE protective loop is the regulation of the ATPase conductivity (Kramer et al., 2003; Avenson et al., 2004). In OE TGase case it seems that differences in ATPase conductivity cannot account for the large effect in qE. Another cause of differences in antenna regulation could be the size of the antenna. By performing similar light activation curves with a known mutant of PSII antenna (ko6 line) we show that 26% decrease in antenna size changes only marginally the activation threshold of qE (**Figure 7A**). Thus antenna size is probably not the reason of different activation of qE. On the other hand it is known that changes in the partitioning of *pmf* can explain different activation rates of qE (Avenson et al., 2005; Ioannidis et al., 2012a). Coexistence of high LEF and low qE in OE TGase indicates that a higher partitioning of *pmf* into Δψ is established in thylakoids. Indeed by deconvoluting the ECS from scattering and xanthophylls cycle contributions we estimated that OE TGase has a 15% higher Δψ/*pmf* (**Table 1**). Unfortunately, our deconvolution procedure although adequate for WT was problematic for most intensities checked in OE TGase mainly due to the very small scattering component. This obstacle stopped us from estimating Δψ/*pmf* under lower light intensities, as well as estimating the sensitivity of antenna down-regulation to ΔpH/*pmf.* The implications of increased TGase activity with respect to the Δψ/pmf perhaps are related to covalent attachment of amines in curtain residues of thylakoids proteins and improve their protonic network in line with the concepts of Dilley and Homann (Johnson et al., 1983; Dilley, 2004).

The emerging role of TGase in chloroplasts is getting clearer after this study. First, it contributes to the regulation of the absorption cross section of PSII. This is in line with early results showing that LHCII, CP29, CP26, and CP24 are substrates of the plastidial TGase (Del Duca et al., 1994) and more recent results showing that TGase is in contact with ATPase and PsbS (Campos et al., 2013, 2014). Second, the polyamine network/metabolism is related to photoprotective qE response. More particularly, increase of putrescine via exogenous putrescine supply increases qE/ΔA535 and increase of TGase activity decreases qE/ΔA535. Third, it affects the functionality of the photosynthetic apparatus by increasing the electric potential difference across thylakoids (present work and Ioannidis et al., 2012b). It is not clear if this effect is due to more polyamines attached to the proteins embedded in the coupling membrane and/or due to release of bound polyamines and elevation of the free pool of polyamines in chloroplasts. Recent studies showed that free putrescine could elevate Δψ in tobacco and *Arabidopsis in vivo* (Ioannidis et al., 2012a). Future experiments will answer whether LHCB1-6 are getting more or less polyaminylated due to TGase overexpression.

## Conclusion

Overexpression of the plastidial transglutaminase from maize increased the activity of transglutaminase in thylakoids of *Arabidopsis*. OE TGase showed a smaller antenna per PSII (-25%), a lower photoprotective qE (-77% at 490 μmol photons m^-2^ s^-1^) due to a higher threshold of qE activation.

## Author Contributions

NI, MS, JT, KK designed research; NI, DM performed research; MS, JT, KK provided materials; NI wrote the manuscript.

## Conflict of Interest Statement

The authors declare that the research was conducted in the absence of any commercial or financial relationships that could be construed as a potential conflict of interest.
