# Supplementary figures and images for: The Over-expression of the Plastidial Transglutaminase from Maize in Arabidopsis Increases the Activation Threshold of Photoprotection
Source: Front Plant Sci. 2016 May 10;7:635. doi: 10.3389/fpls.2016.00635 (PMC4861818; doi:10.3389/fpls.2016.00635)

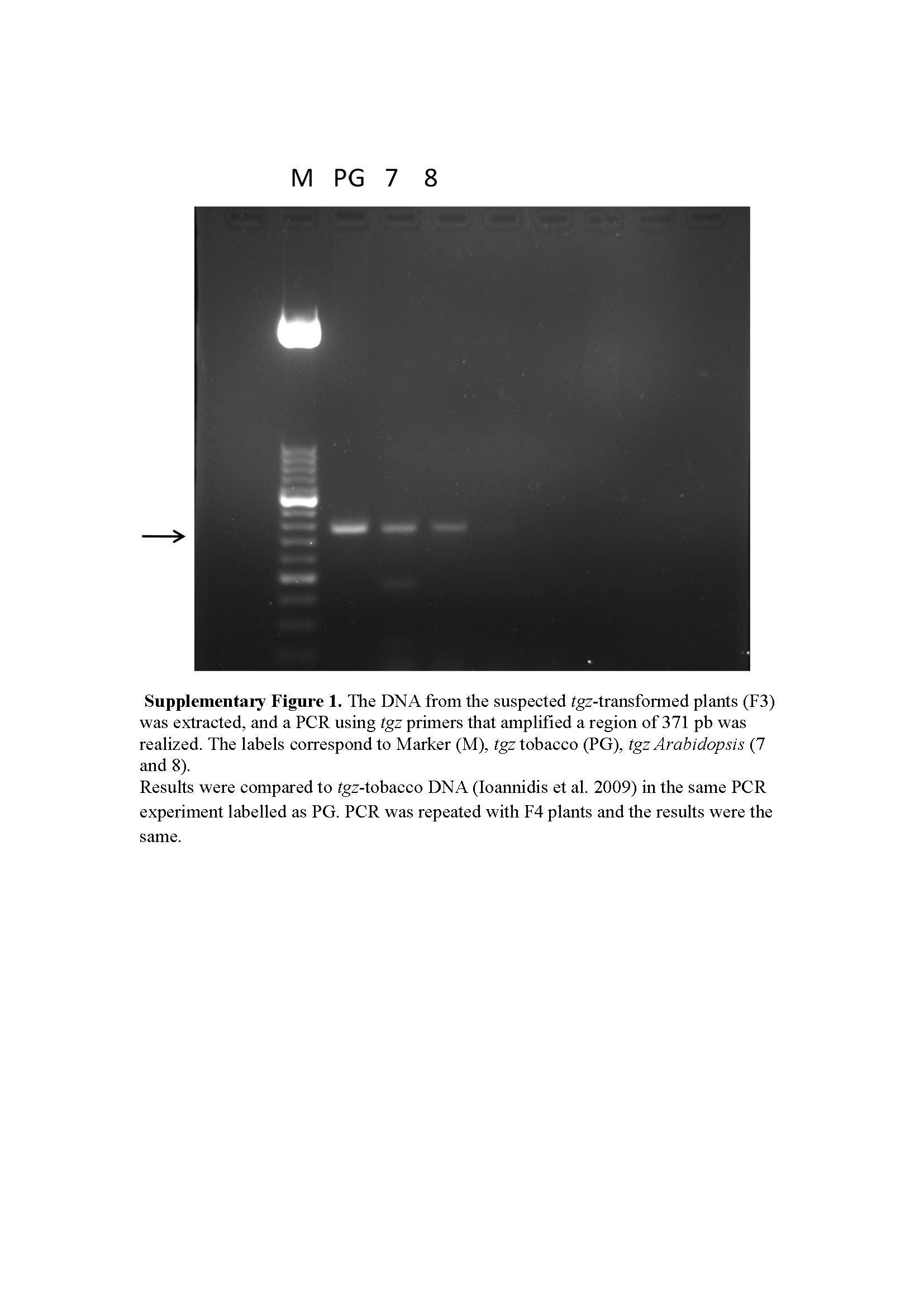

Supplement: Supplementary file 1 [file Image_1.TIFF]

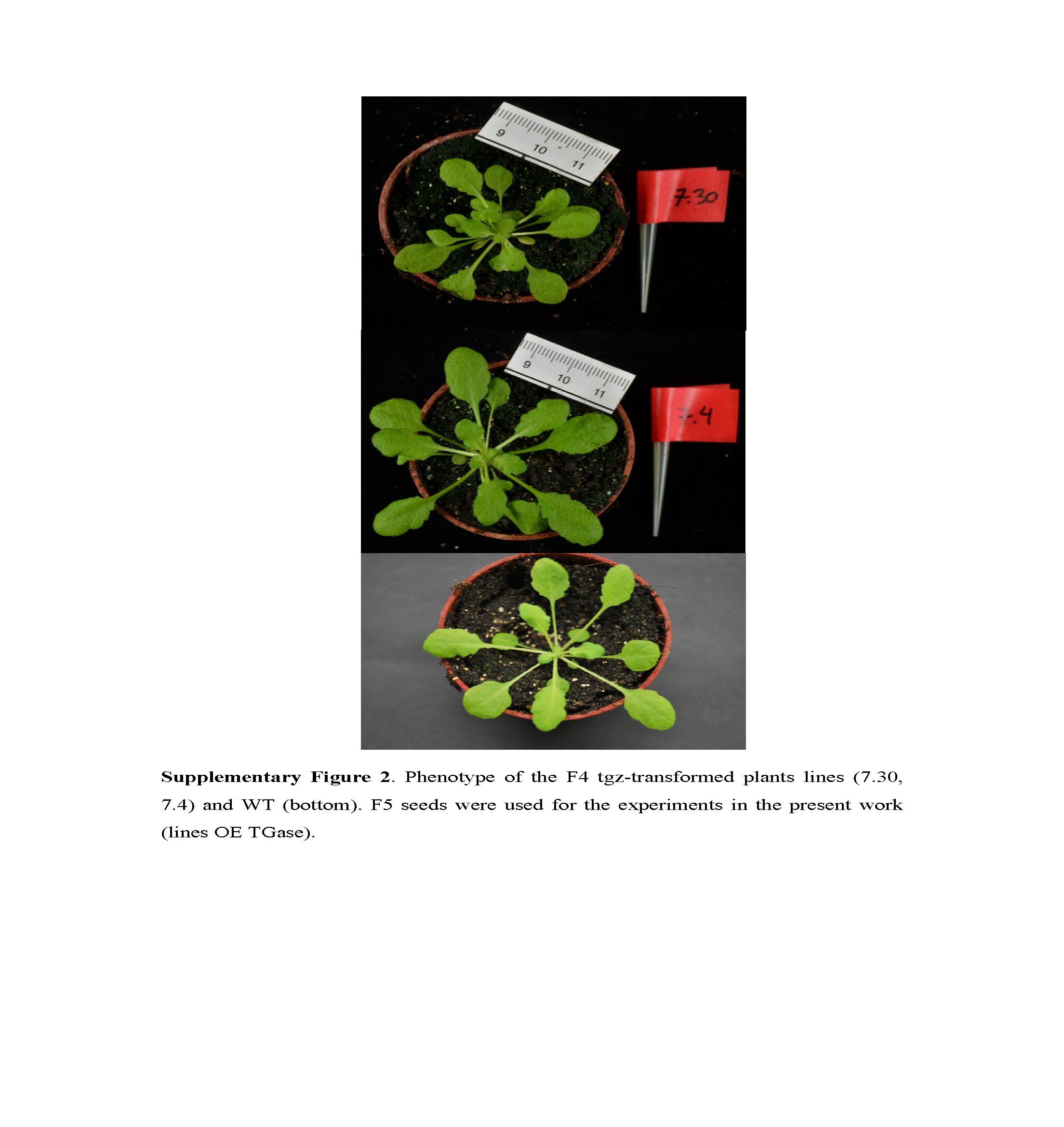

Supplement: Supplementary file 2 [file Image_2.TIFF]
